# Heavy Metal Concentrations of Beeswax (*Apis mellifera* L.) at Different Ages

**DOI:** 10.1007/s00128-023-03779-5

**Published:** 2023-08-20

**Authors:** Nadia M. Hassona, Aida A. Abd El-Wahed

**Affiliations:** 1 Economic Entomology & Apiculture - Plant Protection Department, Faculty of Agriculture (Saba Basha), Alexandria University, Alexandria, Egypt; 2 Department of Bee Research, Plant Protection Research Institute, Agricultural Research Centre, Giza, 12627 Egypt

**Keywords:** Honeybees, Beeswax, Age, Heavy metals, Atomic absorption spectrophotometer

## Abstract

Beeswax is a naturally occurring product that worker bees produce. Beeswax is used in a variety of industries and pharmaceuticals. Humans utilize it extensively in cosmetics, medicinal formulations, and food manufacturing. Beeswax is an essential component of advanced contemporary beekeeping. Beekeepers, in particular, utilize significant amounts of beeswax to make beeswax comb foundation. In its natural condition, beeswax is white, but it becomes yellow then dark in color when it comes into touch with honey and pollen. The ongoing use of wax comb in bee activities (such as brood rearing, storage honey and bee bread), combined with environmental factors such as heavy metal and pesticide residues, resulted in a black color. Because of heavy metals can accumulate in wax for decades, beeswax can be a helpful tool for gathering data on hazardous contaminants in the environment. Because of their lipid-based chemical composition, beeswax combs act as a sink for numerous ambient pollutants as well as poisons when in the hive. The current study aims to measure nine heavy metals and important elements, including iron (Fe), chromium (Cr), zinc (Zn), copper (Cu), nickel (Ni), manganese (Mn), lead (Pb), cadmium (Cd), and cobalt (Co) in beeswax collected in the Behaira governorate region of Egypt between 2018 and 2022. Sample collection was conducted each year in triplicate. The samples were analyzed using an atomic absorption spectrophotometer. The quantity of metals in beeswax at different ages differed significantly. Depending on the wax age, Fe has the highest concentration in the range of 2.068 to 5.041 ppm, while Cd has the lowest ratio at 0.024 to 0.054 ppm from the first to fifth years old of comb age. The findings showed that as beeswax combs aged, the concentration of heavy metals rose. According to the study, it should gradually recycle beeswax combs each year and also adding new foundations.

## Introduction

A naturally occurring honeybee product, beeswax is a highly complex mixture composed primarily of higher fatty acid esters, alcohols, hydrocarbons, proteins, and other minor components (Fratini et al. 2016). In addition to being utilized in the food, chemical, cosmetic, and pharmaceutical industries, beeswax is principally used in beekeeping to manufacture comb foundations (Al-Waili 2005; Amin et al. 2017; Svečnjak et al. 2019; Chaireh et al. 2020). It is fundamental for the honeybee because it is used to build the comb cells that store honey and bee bread. Beekeeping practices and quality depend largely on effective comb management, and beeswax manufacturing techniques have a significant impact on the sensory qualities of the wax (Smith et al. 2017).

Beeswax may be used to detect the presence of metals and pesticides in soil, water, and plants as a bio-indicator of pollution. Due to their unique lipid-based chemical composition, beeswax combs act as reservoir for a variety of environmental pollutants and toxins (Ngat et al. 2020; Ćirić et al. 2021).

Heavy metals can have a variety of hazardous consequences for consumers and bees and depend on how polluted the air, water, and soil (Kast and Kilchenmann 2022; Ullah et al. 2022). Long-term exposure to high levels of heavy metals may cause kidney damage, high lead levels in the blood (Zhang et al. 2012), carcinogenic risks for adults and children (Mao et al. 2019), gastrointestinal problems (Wang et al. 2020), as well as potential implications in impaired fertility (Wang et al. 2020). Heavy metals affect bee development, brood rearing, adult bee lifespan, pollen, nectar, and honey storage, and consumption behaviors (Bromenshenk et al. 1991; Hesketh et al. 2016; Burden et al. 2019).

Because heavy metals can accumulate over a long period of time, beekeepers regularly remove old, dark, and damaged combs during the active beekeeping season and transport them to the craft unit for processing into fresh wax foundations (Svečnjak et al. 2019).When compared to fresh combs, the quality and age of used combs can dramatically improve honeybees workers brood areas, worker population, worker life span, weights of freshly emerged workers and drones, and honey yield (Abd Al-Fattah et al. 2021). Heavy metals are transported to the hive as a result of bee physiological activity or human activity during routine beekeeping procedures (Borsuk et al. 2021). Heavy metal pollution of honeybees, honey, and pollen is extensively established. However, beeswax is rarely studied, most likely because it is not ingested, so it is typically out of the purview of scientific research. Heavy metal concentrations are not regularly tested in beeswax foundation manufacturing. Atomic absorption spectroscopy is extensively method used in assessing heavy metal levels. As a result, it is utilized to determine heavy metals in honeybees and their products (Pohl 2009; Gajger et al. 2016; Adugna et al. 2020).

As part of ongoing research on honeybees and bee products, nine heavy metal elements including the most dangerous ones were detected in the wax combs’ that were collected from locations in the Itay El Barud district of Egypt’s El-Behaira governorate using a validated atomic absorption spectrophotometer method and compared with results of beeswax sample analyses published in the literature (El-Wahed et al. 2021; Khalifa et al. 2021; El-Seedi et al. 2022a, b, c; Salama et al. 2022).

## Materials and Methods

### Samples Collection

**Fig. 1:**
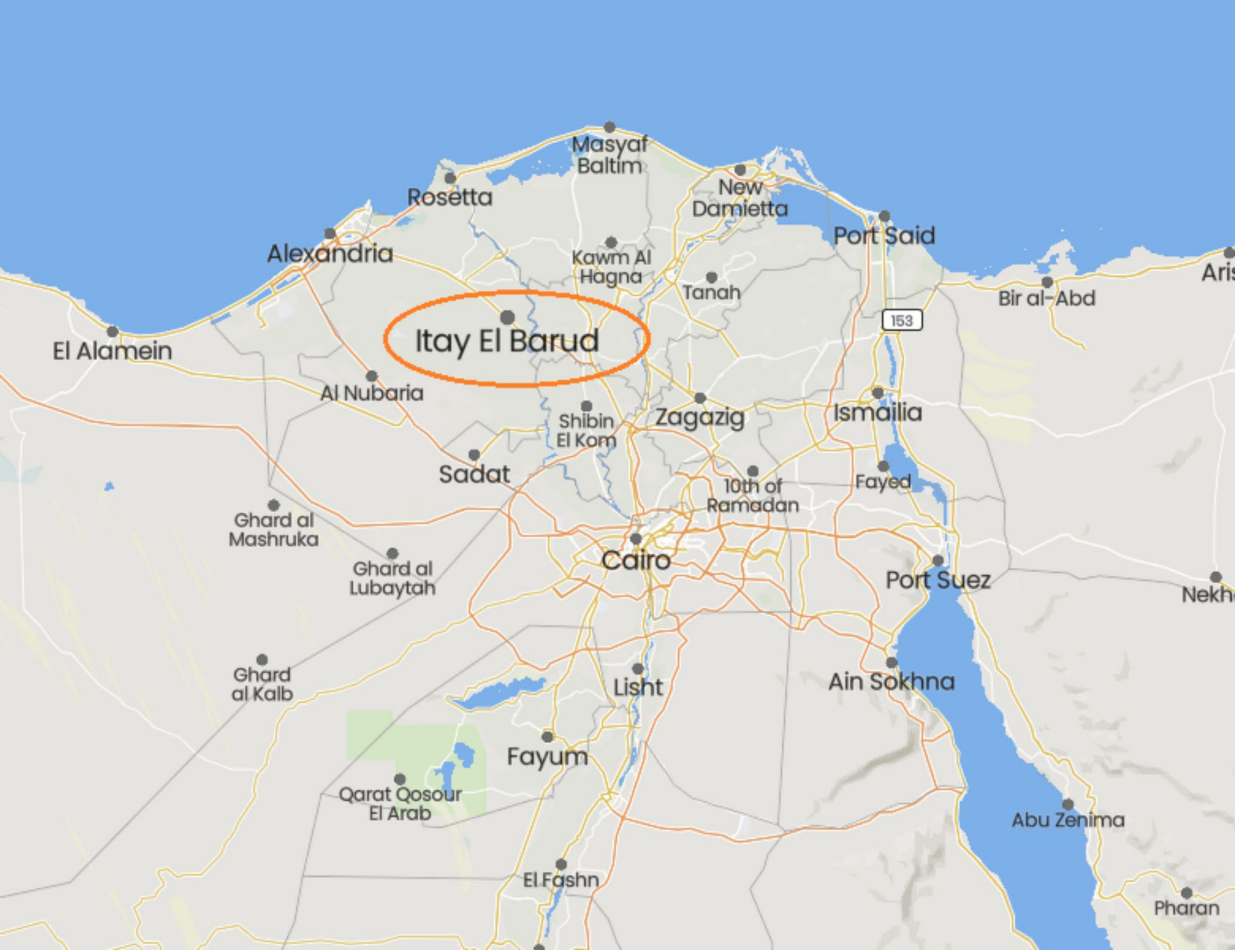
Map with sampling region in El-Behaira governorate, Egypt

The honeybee wax samples were collected from the private apiary in Itay El Baroud region in El-Behaira governorate (Fig. 1, https://mapcarta.com/W312269819/Directions, accessed on November 13, 2022). The apiary is located behind a small road that carries agricultural tractors, cars, and tactics. The honey beeswax samples were collected from five chosen experimental years’ colonies from 2018 to 2022. In addition, from each colony, three honeybee wax frames were chosen, and with the knife, five wax pieces (5 × 5 cm) were randomly taken from each honeybee wax frame, and then saved in a plastic white bag in -5 C^o^ freezer until analysis.

### Samples Analysis for Heavy Metals

Samples were analyzed and determined by an atomic absorption spectrophotometer (Analyst, 400, Perkin Elmer, USA) according to AOAC guidelines (1995) in laboratories of in National Institute of Oceanography and Fisheries (NIOF) as describe herein (Lee 1995). The wax samples were digested then 1 g of each sample was taken and 5 ml of highly purified nitrogenous acid and 2 ml of oxygenated water were added to it, then they were placed in glass test tubes in a water bath at a temperature of 100 °C for about 18 h, until the digestion process was completed. The samples were left for the second day to cool completely, then transferred to volumetric flasks and completed the volume up to 50 ml using deionized water, then the samples were filtered using 0.4 μm filter paper. The samples were kept in polyethylene containers at (-4 °C) until the analysis process. The concentrations of iron (Fe), chromium (Cr), zinc (Zn), copper (Cu), nickel (Ni), manganese (Mn), lead (Pb), cadmium (Cd), and cobalt (Co) in ppm were measured in 15 samples wax using atomic absorption device at different ages of beeswax (Bosancic et al. 2020; Ullah et al. 2022). Limit of detection (LOD) values for the metals were Fe (0.08 mg/l), Cr (0.08 mg/l), Zn (0.01 mg/L), Cu (0.04 mg/l), Ni (0.08 mg/l), Mn (0.025 mg/l), Pb (0.2 mg/l), Cd (0.012 mg/l), and Co (0.06 mg/l).

### Statistical Analysis

The statistical analysis of variance was carried out using the SPSS 26 program by following the one way - ANOVA method, and it was revealed that there were significant differences in the mean concentrations of the studied elements in the beeswax samples, and the statistical significance was estimated at a 95% confidence level.

## Results

### Elements Concentration in Beeswax

The mean concentrations of heavy metals were determined via analysis of wax samples, as listed in Table 1. A statistically significant difference was reported among the samples regarding the levels of heavy metals The mean concentrations of nine elements (Cr, Pb, Cu, Ni, Fe, Zn, Mn, Co, and Cd) were determined using an atomic absorption spectrophotometer. The results showed an influence of wax age on the heavy metal contents, where the mean concentrations of Fe, Cr, Zn, Cu, Ni, Mn, Pb, Cd, and Co were 5.041, 2.728, 2.480, 2.573, 2.547, 1.204, 0.185, 0.054, and 0.054 ppm, respectively, of samples aged 5 years old, while the mean concentrations of samples aged 1 year old were 2.068, 1.008, 0.364, 0.651, 0.890, 0.222, 0.040, 0.024, and 0.027 ppm, respectively. Beeswax samples from the first to fifth years old of comb age show a substantial difference in metal content. Pb, Cd, and Co were reported at the lowest levels in the ranges 0.040 to 0.185, 0.024 to 0.054, and 0.027 to 0.054 ppm, respectively.

**Table 1:** The mean concentrations of heavy metals (ppm) in honey beeswax combs (n = 15)at different ages

Element	Symbol	Beeswax combs aged (years)							L.S.D_0.05_
		**First**	**Second**	**Third**		**Fourth**		**Fifth**	
Iron	Fe	2.068 ± 0.19^c^	2.756 ± 0.11^bc^		3.295 ± 0.57^b^		3.601 ± 0.43^b^	5.041 ± 0.81^a^	0.895
Chromium	Cr	1.008 ± 0.20^c^	1.328 ± 0.41^bc^		1.508 ± 0.49^bc^		2.168 ± 0.44^ab^	2.728 ± 1.05^a^	1.070
Zinc	Zn	0.364 ± 0.01^c^	0.502 ± 0.06^c^		1.585 ± 0.16^b^		1.788 ± 0.24^b^	2.480 ± 0.62^a^	0.559
Copper	Cu	0.651 ± 0.15^c^	0.715 ± 0.15^c^		0.902 ± 0.16^c^		1.930 ± 0.27^b^	2.573 ± 0.27^a^	0.377
Nickel	Ni	0.890 ± 0.17^d^	1.042 ± 0.1^ cd^		1.500 ± 0.36^bc^		2.164 ± 0.46^a^	2.547 ± 0.39^a^	0.593
Manganese	Mn	0.222 ± 0.06^c^	0.365 ± 0.04^c^		0.645 ± 0.18^bc^		0.775 ± 15^b^	1.204 ± 0.38^a^	0.365
Lead	Pb	0.040 ± 0.00^c^	0.043 ± 0.00^c^		0.065 ± 0.02^c^		0.142 ± 0.02^b^	0.185 ± 0.03^a^	0.028
Cadmium	Cd	0.024 ± 00^c^	0.040 ± 0.01^b^		0.043 ± 0.01^b^		0.048 ± 0.01^ab^	0.054 ± 0.00^a^	0.010
Cobalt	Co	0.027 ± 01^c^	0.036 ± 0.01^b^		0.041^b^ ± 0.00		0.048 ± 0.00^ab^	0.054 ± 0.00^a^	0.007

The values represent the mean ± standard deviation (S.D). Significant differences are denoted by different superscript letters (p 0.05). Means in the same row with the same letter are not statistically different at p 0.05

## Discussions

Because beeswax is not directly consumed by people, however, beeswax has significant biological and ecological significance as the honeybee brood nest because the comb cells are where adult bees store their food. Beeswax is a highly effective accumulator of heavy metals, such as Fe, Cr, Zn, Cu, Ni, Mn, Pb, Cd, and Co (Gajger et al. 2019). There is little research on the heavy metal content of beeswax, and there is not enough information on how it affects the quality of the comb (Bommuraj et al. 2019; Ullah et al. 2022).

The aim of the current study was to evaluate the heavy metals content of beeswax at different ages. Beeswax’s heavy metal composition depends on a number of variables, including its geography and beeswax age (Gajger et al. 2019). The wax comb samples were collected from an apiary in the Behaira governorate region of Egypt. The concentrations of heavy metals Fe, Cr, Zn, Cu, Ni, Mn, Pb, Cd, and Co were reported. The wax age had an effect on heavy metal concentrations, as shown in Table 1. Where the highest contents of heavy metals recorded for wax at the 5^th^ year old and the lowest for wax at the 1^st^ year old. Fe, Cr, Zn, Cu, Ni, and Mn were reported highly concentrations compared to Co, Cd, and Pb.

In the current study, the mean content of Fe ranged from 2.068 to 5.041 ppm, which is less than reported by Gajger et al. where the content of Fe ranged from 56.470 to 285 ppm (Gajger et al. 2019). The content of Fe from the various environmental sources was reported within the range of 1.080 to 334 µg/g (Formicki et al. 2013) and 5.972–18,516 mg/g (Aljedani 2020). The higher concentration of Fe may be due to different geographical locations where Fe may be co-occur in areas with similar environmental pollution loads. However, Fe is required not just for haemoglobin formation and oxygen transport but also for the activity of several enzymes. Fe metabolism disorders are among the most frequent illnesses in humans, ranging from anaemia to Fe excess and perhaps neurodegenerative disease. The amount of Fe in your diet depends on the age and gender (Abbaspour et al. 2014).

Cr is one of the 14 most dangerous heavy metals and one of the top priority pollutants, according to the Environmental Protection Agency. Cr is a naturally occurring element that is often used in industrial processes. Humans and other living things require trace levels of the element. Cr is an essential element that has beneficial effects on humans and plays a significant role in blood sugar regulation and lowering. A high dose of Cr can cause a variety of problems, including death in humans where the cytotoxic and genotoxic effects (Speer et al. 2019). The World Health Organization (WHO) set a daily limit of 250 g for Cr (ANS) 2010). In the current study, the content of Cr was 1.008 to 2.728 ppm, based the environmental conditions and regions, the Cr content were different. The presence of Cr accumulated in the wax could be considered an indicator of environmental pollution. The content of Cr ranged from 41.030 to 56.280 ppm (Gajger et al. 2019), 2.016, 2.300, and 3.920 mg/g (Aljedani 2020), 82 to 982 µg/kg (Zafeiraki et al. 2022), 432 µg/kg (Bommuraj et al. 2019), 131.200, 85.770, and 247.600 µg/kg based on the locations of beeswax from three different regions (Ćirić et al. 2021).

A similar pattern was also observed for Zn levels in beeswax, ranging from 1 to 81.200 µg/g (Formicki et al. 2013), 5.707 µg/g (Ullah et al. 2022), 19.699, 6.272, and 0.776 mg/g (Aljedani 2020). Zn is one of the most critical and necessary elements, with major public health implications. Zn is essential for the production of protein and collagen, which aid in wound healing and the maintenance of healthy skin. Growth and developmental delay, sensitivity to infections, delayed sexuality, mental fatigue, and skin toxicity are all possible consequences of the deficiency (Prasad 2020; Lu et al. 2023).

Cu oral intake is usually not harmful to humans. Ingestion of excessive doses of soluble Cu salts can result in severe gastrointestinal discomfort, on rare occasions, and liver damage in vulnerable people exposed repeatedly (Taylor et al. 2020). According to the current work, age of wax influences in Cu content, which ranged from 0.651 to 2.573 ppm, were less significant content than those from different regions that contain high the levels of Cu: 12.8 to 40.93 ppm (Gajger et al. 2019), 1.034, 1.139, 1.913, and 0.095 mg/g (Aljedani 2020), and also 280 to 4244 µg/kg from three regions (Zafeiraki et al. 2022).

Ni is a trace mineral that is necessary for the survival of many animal species, microorganisms, and plants. Adults’ Ni requirements range from 25 to 35 g/day (Anke et al. 1995). Excessive Ni consumption and exposure can result in allergies, cardiovascular and renal illness, lung fibrosis, lung, and nasal cancer. In adults and adolescents, an oral reference dosage of 0.040 mg Cu/kg/day would be protective against acute or chronic toxicity (Genchi et al. 2020; Taylor et al. 2020). Ni is a heavy metal that is detected in wax at different ages, from 1 to 5 years old, at a ratio of 0.890 to 2.547 ppm. The previously published data revealed different concentrations of Ni in beeswax ranged from 194 to 1919 µg/kg (Zafeiraki et al. 2022), 12.170 to 17.830 ppm (Gajger et al. 2019), 15 to 313 µg/g (Formicki et al. 2013), 0.474, 0.598, and 0.678 mg/g (Aljedani 2020) and 0.251 µg/g (Ullah et al. 2022).

Also, Mn content varies based on factors including; geography. The values were 0.182 to 41,904 mg/kg (Zafeiraki et al. 2022), 16.630 to 32.870 ppm (Gajger et al. 2019), 22.200 to 450 µg/g (Formicki et al. 2013), 0.365, 0.501, 1.311, and 0.414 mg/g (Aljedani 2020). Mn is available in low amounts in a range of food sources, providing enough Mn content to support numerous physiological functions in the human body. However, as Mn’s value in a number of sectors grows, so does the risk of overexposure to this transition metal, which can have neurotoxic adverse consequences (Peres et al. 2016).

Pb, Cd, and Co were listed as minor metals in this study. Pb is considered to be one of the most serious environmental toxins, and various studies have shown that it is a cause of health issues. Pb poisoning can induce respiratory, neurological, digestive, cardiovascular, and urinary disorders (Boskabady et al. 2018). When compared to the data displayed in Table 1, the Pd incidence reported in earlier reports and records had a higher concentration (1.230 to 5.430 ppm) (Gajger et al. 2019). Lower Pd levels have been observed to vary between 29.250, 111.800, and 401.100 µg/kg (Ćirić et al. 2021), 255 µg/kg (Bommuraj et al. 2019), 104 to 313 µg/g (Formicki et al. 2013), 0.114, 0.137, and 0.215 mg/g (Aljedani 2020). The concentration of Pb detected in the wax samples depends on the region of the apiaries. Zafeiraki et al. reported the maximum concentrations of Pb from Poša was 3.193 mg/kg while the lowest is 59 µg/kg in Strážske.

Cd is a poisonous heavy metal that is non-essential, an environmental toxin, and harmful even at low concentrations, with no recognized positive role in the human body. Cd consumption has an especially severe impact on fertility and cardiovascular disease (Kumar and Sharma 2019; Genchi et al. 2020). Cd was in the range 0.024 to 0.054 ppm, and the value of Cd was reported by Zafeiraki et al. at 29 to 60 µg/kg (Zafeiraki et al. 2022), 470 to 9870 µg/g (Formicki et al. 2013), 0.059, 0.060, 0.075, and 0.00 mg/g (Aljedani 2020).

Co was also detected at the range of 29 to 129 µg/kg which is less than the data reported in the current study (Zafeiraki et al. 2022). Co is a trace element that is needed for human health and is absorbed through food. As a metal ingredient of vitamin B12, cobalt has a physiologically important function (Cooper, B.A. and Paranchych 1961). However, when consumed in excess, they cause a variety of negative health consequences, most notably neurological, cardiovascular, and endocrine deficiencies (Leyssens et al. 2017).

## Conclusion

The purpose of the current research was to determine whether samples of beeswax taken at various ages may include substantial differences in the values of heavy metal concentrations. Beeswax taken from Egypt’s Behaira governorate was tested for nine heavy metals. According to the study, new beeswax combs only contain fewer amounts of heavy metals. The highest metal concentrations were reported in wax aged 5 years old.

Beekeepers should adopt a new philosophy towards beeswax combs and regularly utilize fresh wax foundation to help decrease heavy metal contamination of honeybee hives and their products, which is also preferable for consumers and the performance of honeybee colonies.
